# What determines employment quality among people living with HIV: An empirical study in China

**DOI:** 10.1371/journal.pone.0243069

**Published:** 2020-12-01

**Authors:** Yunjiang Yu, Zhi Chen, Shenglan Huang, Zhicheng Chen, Kailin Zhang

**Affiliations:** 1 School of International Economics and Trade, Shanghai Lixin University of Accounting and Finance, Shanghai, China; 2 School of Business Administration, Shanghai Lixin University of Accounting and Finance, Shanghai, China; 3 School of Finance, Shanghai Lixin University of Accounting and Finance, Shanghai, China; The Chinese University of Hong Kong, HONG KONG

## Abstract

At the intersection of research areas on health and employment, little attention has been paid on employment quality among people living with HIV (PLWH). The objective of the current study is to identify critical factors and empirically examine their effects on employment quality among PLWH. Based on the social-ecological perspective, we identified negative self-image, workplace discrimination, social support, and policy support as critical factors associated with employment quality among PLWH. Thereafter, a questionnaire survey was conducted to gather information from 339 employed PLWH in China. Hierarchical regression analyses were further performed to analyze the effects of the identified factors on employment quality among PLWH. We obtained three main findings. First, negative self-image and workplace discrimination are detrimental to employment quality among PLWH; whereas social support and policy support are conducive to their employment quality. Second, older, male, and highly educated PLWH can better leverage the undesirable effects of negative self-image and workplace discrimination on employment quality compared with their peers. Third, male, and highly educated PLWH can better utilize social support and policy support to advance employment quality compared with their peers. However, the employment quality effects of the identified factors did not differ by marital status. Our findings provided some useful implications for PLWH, employers, community service providers, and policy makers to promote employment quality among PLWH.

## Introduction

Employment participation has been proven beneficial for people living with HIV (PLWH), because it helps to promote healthy behaviors, such as leading to interpersonal contacts, learning new skills, providing social identity, and revitalizing their own targets [1–3]. In view of the benefits to maintaining active employment for PLWH, many countries have developed supportive employment services whereby the employment rate of PLWH has been greatly raised. For example, the Royal Free HIV Department in the UK reported that 74% of PLWH were in some type of employment [4, 5]. However, alertness has increased that maintaining and enhancing the employment status among PLWH will be insufficient if the works are not psychosocially or physically sustainable for PLWH [6]. Therefore, the concerns of policy makers and researchers in recent years have shifted from finding employment per se to enhancing employment quality among PLWH [7].

The literature has documented that employment quality reflects multidimensional phenomena consisting of two components, namely employment relations and employment conditions [6, 8]. Specifically, employment relations concern with the power relations between employees and their employer on the communicating and signaling mechanisms from employer to employees [6, 9]. Employment conditions refer to the impersonal conditions of employment such as contracts, wages, and working hours [9, 10]. As the life expectancy of PLWH continues to increase, employment quality is now becoming an important issue for the sustainability of their employment [11]. Accordingly, it is important to identify the factors and their roles in the development of employment quality.

However, this branch of research still involves two main shortcomings. First, although a range of factors are highly related to employment status among PLWH including demographic characteristics, psychological and physical health status, workplace discrimination, and social service linkage, a paucity of research has examined their relationships with employment quality [12, 13]. Indeed, some scholars have pointed out that the relationships may differ with employment quality [14]. For example, there is a consistent inverse association between depression and employment status, whereas PLWH with poor quality jobs report similar levels of depression as those who are unemployed [15]. Therefore, our knowledge remains incomplete on the relationships between potential factors and employment quality among PLWH.

Second, few studies on employment among PLWH have been conducted in developing economies, as most studies of this type were mostly found in the context of developed Western economies [6, 16]. Indeed, the situation can differ in developing economies [11]. For instance, following the guidance of UK government on confidentiality, HIV status should not be disclosed by health-care professionals without the employee’s express consent. Nevertheless, PLWH are disqualified in China when they work in public places [5, 17]. Consequently, 15% of PLWH in China reported that their HIV status was reluctantly disclosed by occupational health departments [17]. Therefore, it is unclear whether the employment quality implications of potential factors are applicable to developing economies given the significant differences in institutional environment between developed and developing economies.

Using the social-ecological perspective, we address the two weaknesses by identifying multiple levels of embedded factors associated with employment quality [14, 18]. The advantage of the social-ecological perspective lies in that it recognizes the individual-, organizational-, community-, and policy-level factors collectively influence the behaviors of PLWH [14, 19]. Thus, the social-ecological perspective has been widely applied in understanding multilevel intervention on PLWH [20–22]. To be specific, we identify negative self-image, workplace discrimination, social support and policy support as the individual-, organizational-, community- and policy-level factors, respectively. The literature has highlighted the importance of negative self-image, workplace discrimination, social support and policy support among various potential factors associated with employment quality [5, 23, 24]. For example, workplace discrimination can elevate the degree of stress among PLWH, which was of concern for the employment quality among PLWH [4, 25]. Similarly, failure to secure social support might also mitigate the employment outcomes among PLWH [26].

In sum, the current research first builds on the social-ecological perspective in identifying different level factors associated with employment quality among PLWH, namely, negative self-image, workplace discrimination, social support and policy support. Then, drawing on the dataset of 339 survey informants in China, we empirically examine the effects of the identified factors on employment quality among PLWH. By emphasizing the two aspects of efforts, the current research not only theoretically enriches the literature on identifying the factors associated with employment quality among PLWH, also provides empirical evidence on the identified factors that may influence employment quality among PLWH in China.

## Materials and methods

### Participants and procedures

We conducted our research by employing the questionnaire survey. First, we adopted the Delphi method [27, 28] to invite three researchers from the research fields of health, employment, and applied psychology to review the survey. In the first roundtable discussion, the researchers reached a consensus about which domains and items to be included in the questionnaire to ensure that this survey would not have any negative influence on the respondents. In the second roundtable discussion, the researchers checked the clarity and relevance of items to guarantee the content validity of the instruments. After the two roundtable discussions, we ensured that our research question, context and constructs have been defined correctly, and also the semantics and grammars of the items have been clarified. Second, the questionnaire survey was approved by the Ethics Committee of Shanghai Lixin University of Accounting and Finance. We obtained a written informed consent (Identification Code 2018LL-12-001) from the school when we assured the participants from the survey were confidential, and our purpose was exclusively for academic research. Third, an online questionnaire survey was conducted at the local Centre for Disease Control and Prevention (CDC) in Shanghai, Guangdong, and Zhejiang provinces of China from December 2018 to February 2019. This convenience sampling method is common among empirical studies on health and employment, because the local CDCs are vital to deliver care, counseling, and support services to PLWH, and thus know most information on PLWH in the local areas [29–31]. Then, the targeted sample was chosen as those employed who were diagnosed with HIV and voluntarily collaborated with the researchers [32]. Consistent with previous studies [26], those with severe mental and physical disabilities or had difficulty in reading the survey were excluded. With the help of the local CDCs, the online questionnaire was distributed via the Sojump software which is popular in China [33]. Finally, a total of 423 questionnaires were received. After deleting the missing values and reviewing the reverse items we have set, 339 were valid ones.

### Measurement

To ensure the validity and reliability, the measurement scales of our constructs were adopted from the existing studies. All the items were measured with five-point Likert scales, scoring from 1 (i.e., extremely disagree) to 5 (i.e., extremely agree).

*Negative self-image* was measured by five items adapted from Friedman et al. [34] and Berger et al. [35]. The participants were asked to evaluate their senses of guilt and shame including the feelings of being inferior to others, and liking a bad person owing to HIV-infection. Example items were “HIV-infection makes me feel unclean” and “I feel I'm inferior to others because I have HIV”.

*Workplace discrimination* was measured by four items adopted from Wagener et al. [24]. The participants were asked to rate the perceived consequences of coworkers knowing her/his HIV-infection, such as losing friends, avoiding touching her/him, and the feeling of regreting for such information disclosure. Sample items included “Others avoid shaking hands with me when they know I’m HIV positive” and “Telling others has been a mistake”.

*Social support* was measured by four items adapted from Stewart et al. [30]. The participants indicated the degree to which they receive directive support from community service providers. Examples of the items were “Give me clear advice on how to handle employment-related problems” and “Solve employment-related problems for me”.

*Policy support* was measured by three items from Huang et al. [36]. The participants were asked to assess how they perceive supportive employment policies from the government. Sample items included “The government has developed sound policies to train, attract and encourage me” and “The government has established a stable human resources management system for me”.

*Employment quality* was measured by seven items developed from Van Aerden et al. [6]. These items included two dimensions, namely, employment conditions and employment relations. Specifically, employment conditions reflect the participants’ perceptions of contract, rewards, right protection, working hours. A sample item is “My employment is in stability”. Employment relations reveal the participants’ perceptions of employment opportunities, and interpersonal power relations. A sample item is “My training opprtunities are limited”.

*Control variables* were chosen as demographics, because demographics can influence PLWH’s access to government- and community-based programs supporting employment [24]. These included: age in groups (0 = 20 years and below, 1 = 21–30 years, 2 = 31–40 years, 3 = 41 years and above); gender (0 = female, 1 = male); marital status (0 = unmarried, 1 = married); and education level (0 = junior school and below, 1 = high school, 2 = college, 3 = undergraduate and above).

### Analytic strategy

A series of hierarchical regression analyses assessed the relationships among negative self-image, workplace discrimination, social support, policy support, and employment quality. In the first step, demographics were entered as a block in the regression analyses. In the second step, negative self-image, workplace discrimination, social support, and policy support, or their interactions with demographics were entered as a block. The variance inflation factor (VIF) scores were all smaller than two, which means that the multicollinearity among the variables is not serious [37].

## Results

### Descriptive results

Table 1 showed that among the 339 respondents, 50.1% were male and 49.9% were female. Age distribution was unevenly, and skewed to the age of 21–30 years (23.0%), 31–40 years (23.9%), and 41 years and above (43.7%). Marital status distribution showed that the majority of the respondents were married (75.8%). Regarding the education level of the respondents, 81.7% of them have a college degree and above.

**Table 1:** Sample demographic (n = 339).

Variables	Number	Percentage
**Gender**		
Female	169	49.9%
Male	170	50.1%
**Age**		
20 years and below	32	9.4%
21–30 years	78	23.0%
31–40 years	81	23.9%
41 years and above	148	43.7%
**Marital status**		
Unmarried	82	24.2%
Married	257	75.8%
**Education level**		
Junior school and below	15	4.4%
High school	47	13.9%
College	126	37.2%
Undergraduate and above	151	44.5%

### Measurement evaluation

Before hierarchical regression analyses, confirmatory factor analysis was performed to test the reliability and validity of negative self-image, workplace discrimination, social support, policy support, and employment quality. First, the Cronbach’s alpha values of these constructs ranged from 0.82 to 0.90, which exceeded the standard of 0.70. The composite reliability scores ranged from 0.88 to 0.93, which also surpassed the conventional threshold of 0.70. Thus, an acceptable level of reliability was supported. Second, all the factor loading scores were larger than 0.70, which exceeded the benchmark of 0.60. The average variance extracted (AVE) scores were above the criterion of 0.50. Hence, a good convergent validity was supported.

### Testing the main effects on employment quality among PLWH

Table 2 showed the regression results of the main effects. In Model 1, the four control variables, namely, age, gender, education level, and marital status have non-significant effects on employment quality among PLWH. Model 2 revealed that negative self-image had a significantly negative effect on employment quality among PLWH (*β* = -0.51, *p* ≤ 0.01). Models 3 demonstrated that workplace discrimination had a significantly negative effect on employment quality among PLWH (*β* = -0.65, *p* < 0.01). As depicted in Model 4, we found that social support had a positive impact on employment quality among PLWH (*β* = 0.45, *p* < 0.01). Lastly, Models 5 presented that policy support has a positive impact on employment quality among PLWH (*β* = 0.21, *p* < 0.01).

**Table 2:** The results of the main effects on employment quality among PLWH.

Variables	Model 1	Model 2	Model 3	Model 4	Model 5
Age	0.07	0.04	0.03	0.10	0.07
Gender	-0.06	-0.05	-0.07	-0.10	-0.08
Education level	0.02	0.05	-0.01	-0.01	0.01
Marital status	-0.10	-0.06	-0.04	-0.08	-0.08
Negative self-image		-0.51**			
Workplace discrimination			-0.65**		
Social support				0.45**	
Policy support					0.21**
R^2^	0.01	0.27	0.43	0.22	0.06
F value	1.13	24.68**	49.49**	18.23**	3.86**

Note:

**p < 0.01

*p < 0.05.

### Testing the interactive effects on employment quality among PLWH

Table 3 showed the regression results of the interactive effects. In Model 1, we found that the interactions between age and negative self-image (*β* = 0.10, *p* < 0.05), and between age and workplace discrimination (*β* = 0.11, *p* < 0.01) were both significantly related to employment quality among PLWH. However, the coefficients of the interactions between age and social support (*β* = 0.08, *n*.*s*.), and between age and policy support (*β* = 0.08, *n*.*s*.) were non-significant. In Model 2, the interactions between gender and negative self-image (*β* = 0.21, *p* < 0.01), between gender and workplace discrimination (*β* = 0.11, *p* < 0.01), between gender and social support (*β* = 0.16, *p* < 0.01), and between gender and policy support (*β* = 0.07, *p* < 0.05) were all significantly related to employment quality among PLWH. Similarly, as depicted in Model 3, the interactions between education and negative self-image (*β* = 0.11, *p* < 0.01), between education and workplace discrimination (*β* = 0.12, *p* < 0.01), between education and social support (*β* = 0.23, *p* < 0.01), and between education and policy support (*β* = 0.08, *p* < 0.05) were all significantly related to employment quality among PLWH. However, none of the interaction terms in Model 4 passed the 5% significance level test. It means that the effects of the four factors on employment quality regardless of whether PLWH were married or unmarried.

**Table 3:** The results of the interactive effects on employment quality among PLWH.

Variables	Model 1	Model 2	Model 3	Model 4
Age * Negative self-image	0.10*			
Age * Workplace discrimination	0.11**			
Age * Social support	0.08			
Age * Policy support	0.08			
Gender * Negative self-image		0.21**		
Gender * Workplace discrimination		0.11**		
Gender * Social support		0.16**		
Gender * Policy support		0.07*		
Education level * Negative self-image			0.11**	
Education level * Workplace discrimination			0.12**	
Education level * Social support			0.23**	
Education level * Policy support			0.08*	
Marital status * Negative self-image				-0.04
Marital status * Workplace discrimination				0.01
Marital status * Social support				0.06
Marital status * Policy support				-0.05
Control variables	Yes	Yes	Yes	Yes
R^2^	0.57	0.59	0.60	0.54
F value	35.41**	39.79**	40.79**	32.30**

Note: Control variables include age, gender, education level, marital status, negative self-image, workplace discrimination, social support, and policy support.

**p < 0.01

*p < 0.05.

## Discussion

Our empirical results showed that the four factors guided by the social-ecological perspective were significantly related to employment quality among PLWH. Specifically, negative self-image and workplace discrimination were negatively related to employment quality among PLWH, whereas social support and policy support were positively related to employment quality among PLWH. The results mean that the stronger the negative self-image and workplace discrimination against PLWH, the worse their employment quality would be. By contrast, the more support PLWH receive from the community and government policy, the better their employment quality would be. These findings are similar with previous studies. For example, Zhang et al. [38] argued that PLWH who experience discrimination are likely to have worse physical and psychological health status (e.g., distress and anxiety), which are highly related to inferior employment outcomes. Analogously, Su et al. [26] asserted that social support can help reduce negative psychosocial consequences of the HIV experiences, thereby benefiting for superior employment outcomes.

While earlier research has investigated potential factors associated with employment outcomes [39], scarce research has examined the boundary conditions of these factors. In this study, we examined whether the effects of negative self-image, workplace discrimination, social support, and policy support on employment quality among PLWH differ by demographics. As for age, although previous studies have investigated age differences in perceived psychosocial distress, and workplace discrimination [25], there has been little attention on age differences in psychosocial distress, and workplace discrimination associated with employment quality. We first found that older PLWH can better leverage the undesirable effects of negative self-image and workplace discrimination on employment quality. It may be explained that older PLWH are more likely to use adaptive coping strategy in dealing with burden from negative self-image and workplace discrimination compared with their younger peers [38].

Gender difference was found to be significant in the relationships between the identified factors and employment quality among PLWH. Specifically, our result demonstrated the buffering effect of male on the relationships between negative self-image and employment quality, and between workplace discrimination and employment quality. Also, our result indicated that male can better utilize support from community service providers and government policy for boosting employment quality. These results may be due to sociocultural norms on gender roles in Chinese society. Zhou [40] argued that the norms of womanhood (i.e., being a good woman) continue to have a profound influence on Chinese women’s construction and reconstruction of self-worth or desired identity. Such a gender inequality rooted in sociocultural norms would shape men’s and women’s attitudes to, and experiences of, care receiving or care utilization [41, 42]. For example, the Chinese traditional ideal of womanhood call in HIV-infected woman would impediment them make full use of communal and institutional care resources.

Educational gradient was also significant for the effects of the identified factors on employment quality among PLWH. Specifically, our result suggested that higher education would attenuate the negative effects of negative self-image and workplace discrimination, but amplify the positive effects of social support and policy support. This result is consistent with prior findings. For instance, Rzeszutek [43] argued that higher education an important personal property in coping with HIV-related psychological burden among PLWH, and protecting them from workplace discrimination. Similarly, Chou & Choi [25] noted that highly educated PLWH are capable of accessing and utilizing resources for improved employment outcomes.

### Theoretical implications

This study has two main theoretical contributions. First, following the emerging trends of HIV and employment literature from employment status to employment quality among PLWH [7], this study draws on the social-ecological perspective to identify negative self-image, workplace discrimination, social support, and policy support as critical factors from multiple layers [14]. Hence, this study extends the context-specific applications of the social-ecological perspective to employment quality among PLWH. Moreover, prior research has argued that PLWH rarely access employment-related services [44] which have been more established for people living with other chronic diseases such as stroke rehabilitation [45], cardiac [46], and pulmonary [47]. Accordingly, some scholars have called for more research to identify HIV-specific complex conditions and tailor employment enhancement recommendations for PLWH [48]. To answer these calls, this study also represents a comprehensive scheme of the factors associated with employment quality among PLWH for advancing the research fields of HIV and employment.

Second, as opposed to most existing frameworks developed and examined primarily in developed Western economies [7], the current paper focuses on employment quality among PLWH in the context of China. As McGoldrick [5] stated, Chinese culture for treating PLWH is significantly different from those in developed Western economies. For example, institutional arrangements for protecting PLWH in developed Western economies are strong and work smoothly, so that their role becomes nearly invisible and fades away as “background” conditions for employment quality among PLWH [23]. When institutional arrangements malfunction in developing economies, their defects becomes evident [5, 26]. Therefore, our study extends the established research on HIV and employment quality in developing economies, as represented by China.

### Practical implications

This study also has some practical implications. First, our result suggests that PLWH who have strong feeling of shame and guilt or face bias related to HIV, will encounter additional barriers to employment quality. This result heightens the urgency to meticulously understand the employment quality implications of related individual- and organizational-level factors. One the one hand, PLWH should enhance their capability to cope and persevere in the face of these psychological burdens. One the other hand, employers must realize that the reduction of workplace discriminations will enhance employment quality among PLWH. Thus, employers should assist in eradicating intra-organizational barriers in interpersonal communications. For example, employers should ensure that workplace institutions for preventing HIV discrimination are provided, monitored and strictly followed.

Second, our result indicates that supports from community service providers or the government are associated with employment quality among PLWH. With this in mind, community service providers can receive additional training on stress management services [26, 49] or trauma-informed approaches [3] to more effectively serve marginalized PLWH who are at increased risk of psychological burden. Moreover, government agencies and community service providers can collaborate to address the employment quality needs of PLWH. For example, the Department of Labor can collaborate with the Health and Human Services to develop, disseminate, and implement the service provisions about employment services and resources.

### Limitations and future work

There are some limitations that provide opportunities for future work. First, our study has been hampered by the micro-level measurement scales of employment quality. Despite its advantage of grasping two important aspects (i.e., employment relations and employment conditions) which actually occur in the workplace, employment quality contains not only the characteristics of individual jobs such as personal wages and rights, but also the characteristics related to macro labor market such as social protection coverage, the overall economy performance, and productivity [16, 50, 51]. Future research should prefer comprehensive measures of employment quality, as well as more objective indicators of working hours, job safety, job autonomy, and promotion opportunities. Second, this study used the cross-sectional data, and thus, was lack of longitudinal data. It makes us difficult to infer the causal relationships between the variables. A longitudinal design should be used in the future to investigate the dynamic effects of various variables on employment quality among PLWH. Third, the Chinese sample dataset may restrict the applications of our findings in other national contexts. Future studies can obtain samples from multi-national organizations, and focus on cross-cultural comparisons of the relationships among the variables. Lastly, this study adopted the self-reported measurement, which may cause social desirability and memory biases. Future research can adopt other-rated or more objective data.

## Conclusions

This study empirically examined the identified factors associated with employment quality among PLWH in China, namely, negative self-image, workplace discrimination, social support, and policy support. First, we found that negative self-image and workplace discrimination were negatively related to employment quality among PLWH, whereas social support and policy support were positively related to employment quality among PLWH. Second, we found that older, male, and highly educated PLWH can better leverage the undesirable effects of negative self-image and workplace discrimination on employment quality compared with their peers. Third, we found that male, and highly educated PLWH can better utilize social support and policy support to pursue superior employment quality. Our results enrich the health and employment literature by providing empirical supports for multilevel intervention on PLWH. That is, PLWH should alleviate negative self-image, employers should eradicate workplace discrimination against PLWH, community service providers should improve employment-related services for PLWH, and policy makers should tailor supportive employment policies to advance employment quality among PLWH.
